# Clinical Considerations in Initial Evaluation and Treatment of Hardhead Catfish Spine Puncture Wounds

**DOI:** 10.1155/2021/8841234

**Published:** 2021-03-09

**Authors:** Hannah Gordon, Benjamin J. Cousins, Mark A. Newberry, David A. Farcy

**Affiliations:** ^1^Department of Emergency Medicine, Mount Sinai Medical Center, Miami Beach, Florida, USA; ^2^South Florida Hand Surgery, Miami Beach, FL, USA; ^3^Department of Emergency Medicine & Critical Care, Herbert Wertheim College of Medicine, Florida International University, Miami, Florida, USA

## Abstract

A 17-year-old male presented to our ED complaining of pain and swelling at the base of the first metacarpal after attempting to remove a catfish from his fishing line 12 hours prior to arrival. Radiographic images demonstrated a foreign body (FB), which was detectable by ultrasound. Hand surgery was consulted and took the patient to the operating room for exploration and removal of two serrated radiopaque catfish spines that were deeply embedded in the left thumb. *Conclusion*. Penetrating injury from hardhead catfish (*Ariopsis felis*) spines can cause hidden FB, envenomation, infection, and secondary damage to nearby structures. Imaging should be done for these patients to ensure they obtain timely and complete extraction of the venomous structures. Surgery should be consulted for operative management to avoid damage on removal of the catfish spine remnants.

## 1. Introduction

The saltwater catfish is more formally known as the hardhead catfish (*Ariopsis felis*). Its natural habitat is between the Northwest Atlantic and the Gulf of Mexico. They are commonly found near the Florida shoreline. They are considered a nuisance to fisherman since they are attracted to most types of bait and are often accidentally caught despite their undesirable taste. They have six long barbels surrounding their mouth and chin to help them catch prey and sharp, serrated, mucous-covered pectoral, and dorsal spines. These spines stand erect when the fish feels threatened to help protect it from would-be predators [1]. They contain an integumentary sheath of venom that, when released, can cause a severe localized inflammatory reaction that may spread systemically. For most people, the venom can be neutralized by immersing the wound in a warm water bath for 30 minutes but is most concerning due to its high association with secondary infections, especially in immunocompromised patients [2, 3]. Puncture wound by these spines can penetrate easily and is serrated such that they can be very difficult and even damaging if not removed appropriately. There have been only a few case reports in specialty journals reporting these injuries, including a penetrating renal injury and punctures to the feet and hand [2–6].

## 2. Case Report

A 17-year-old male presented to our ED complaining of stinging pain and swelling at the base of the first metacarpal after attempting to remove a catfish from his fishing line 12 hours prior to arrival. As per the father, the patient was a high school baseball star being recruited by several Major League Baseball teams. The patient and his father were concerned for any future disability to his hand that could threaten his career. The patient stated he had an initial stinging sensation upon contact and felt minor relief after handwashing and acetaminophen, but the pain returned and became progressively more severe overnight with associated throbbing, edema, and erythema. The patient had no history concerning for immunocompromise, was fully vaccinated, and denied signs of systemic infection including fevers, chills, nausea, vomiting, or diffuse rash.

**Figure 1:**
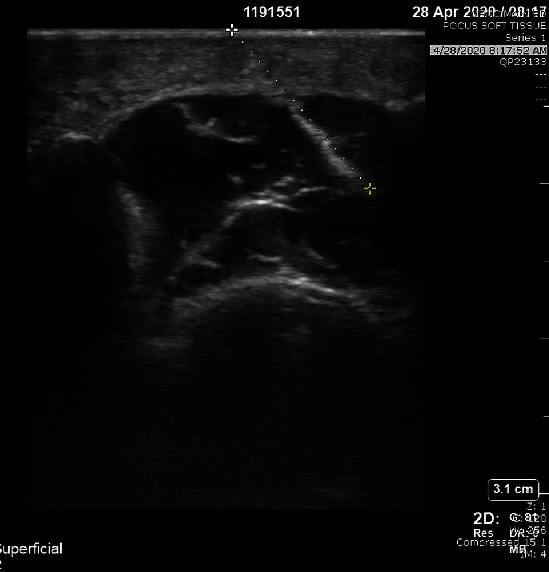
Initial point of care ultrasound demonstrates a thin linear hyperechoic structure with soft tissue swelling edema in the thenar region of the left hand.

**Figure 2:**
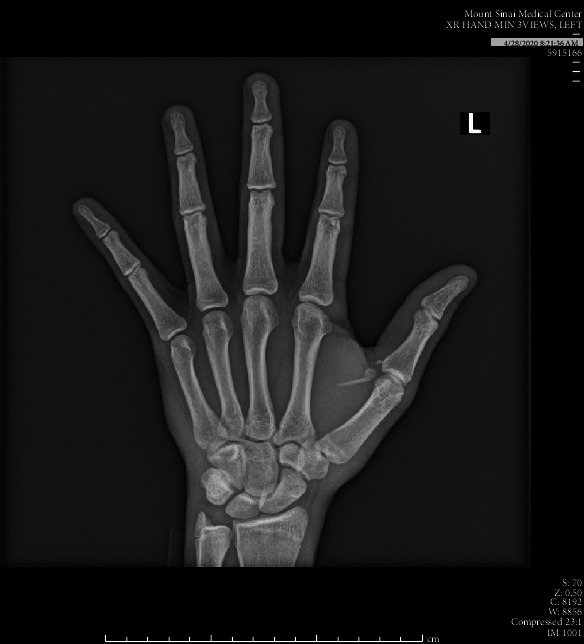
AP radiograph of the left hand demonstrates a 1.7 cm linear radiodensity in the thenar region of the left hand which appears extraosseous, compatible with a foreign body. There is no other acute osseous abnormality.

**Figure 3:**
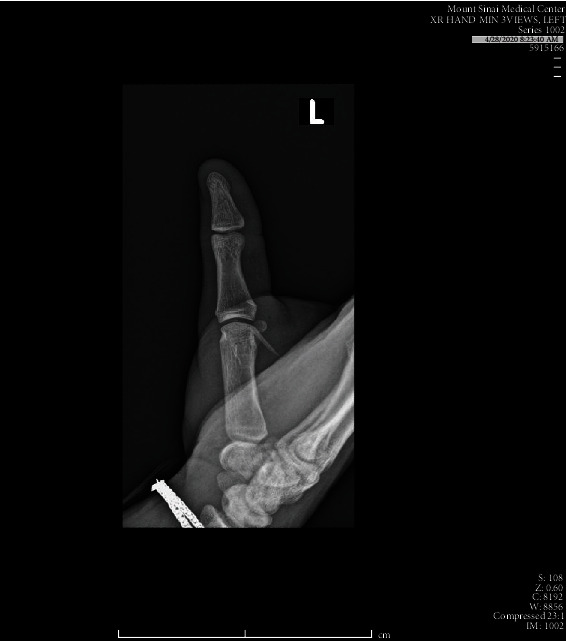
Additional coned-in view of the left thenar region demonstrating a linear foreign body.

**Figure 4:**
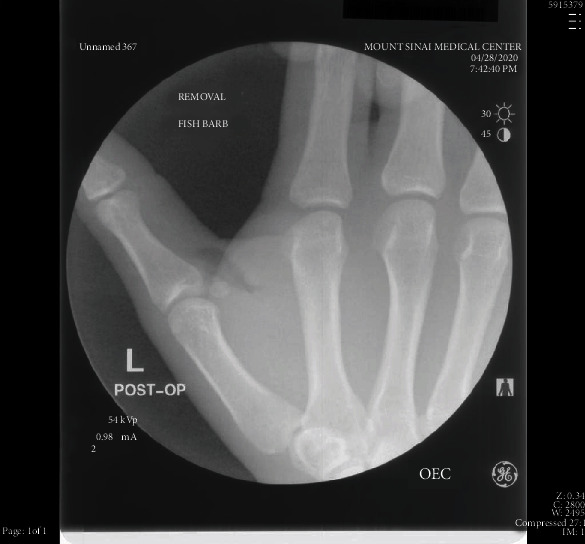
Subsequent fluoroscopic spot view of the left hand demonstrated no residual foreign body. There is some post procedural subcutaneous gas in the area from recent surgical removal.

**Figure 5:**
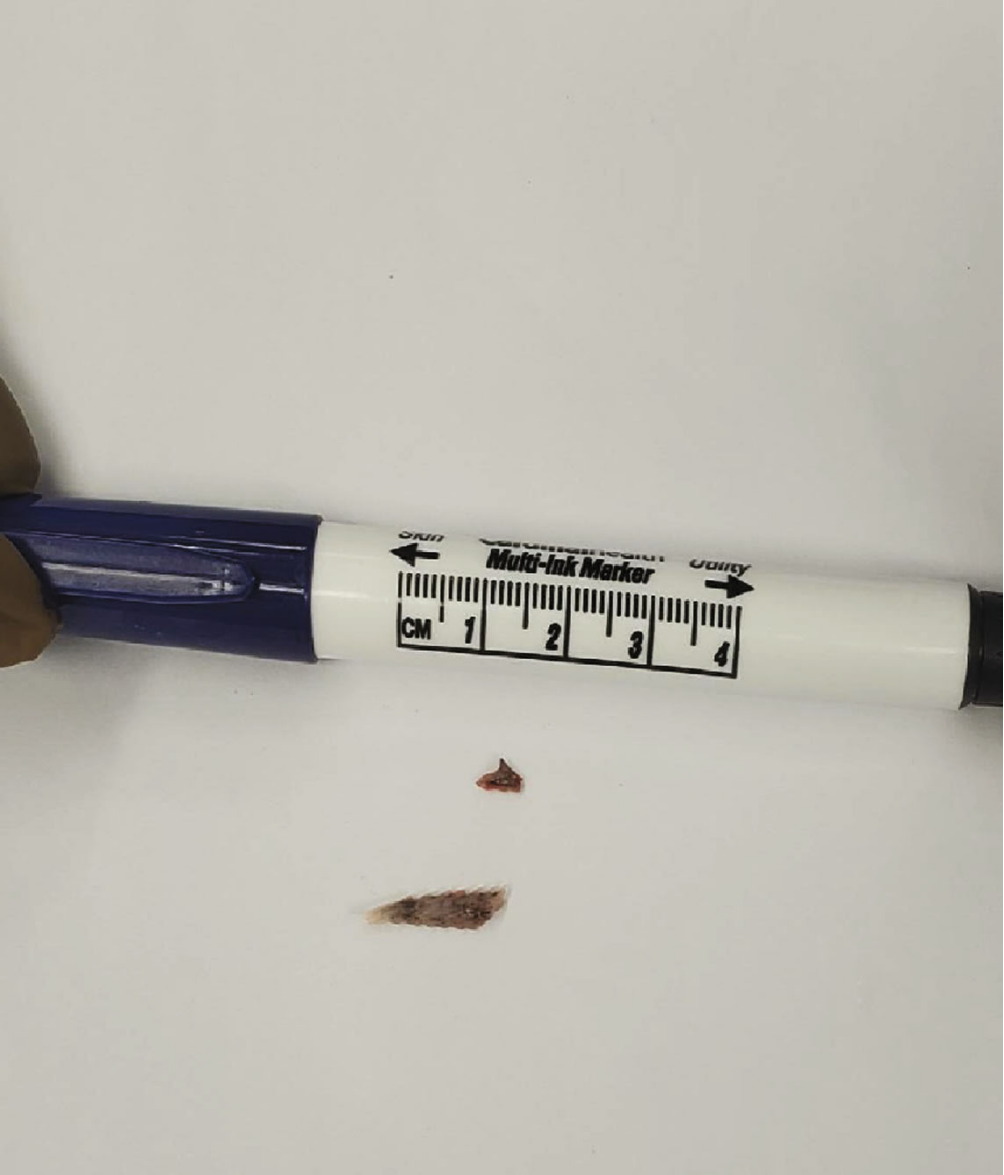
Dorsal spines of hardhead catfish (Ariopsis felis).

Upon presentation, the patient was afebrile with normal vital signs. Exam was significant for 2 cm of edema and erythema along the base of the 1^st^ metatarsal with overlying coagulated blood at puncture site and intact range of motion. Labs demonstrated a leukocytosis of 11.96 and 82.1% neutrophils. Attempt was then made to visualize a foreign body under ultrasound which demonstrated a thin linear hyperechoic structure with soft tissue edema in the thenar region of the left hand (Figure 1). X-rays demonstrated a 1.8 cm and 0.8 cm radiopaque foreign body within the soft tissue at the ulnar margin of the first metacarpal (Figures 2 and 3). Hand surgery was consulted and took the patient to the operating room for exploration and removal of two serrated radiopaque hardhead catfish spines that were deeply embedded in the left thumb. The first was located along the flexor tendon, and a second spine was located buried within the adductor pollicis muscle. Careful operative dissection under loupe magnification was used to ensure preservation of the neurovascular bundles, flexor tendon, pulley system, and thenar musculature. Once the serrated spines were located, gentle manipulation was used to free and release them from the surrounding tissues without causing additional damage (Figures 4 and 5). The patient was prophylactically treated with antibiotics for prevention of secondary infection.

## 3. Discussion

Initially, along with history, radiographs may be adequate for the identification of a fish bone as a foreign body, but generally have low sensitivity (25-39%) [7]. Soft tissue swelling or gas may be the only sign of a foreign body especially with smaller fragments and with some species of fish bones which may not be adequately radiopaque [7, 8].

Ultrasound correlates well with visibility and radio-opacity on radiographs. Ultrasound is especially helpful in detecting superficial foreign bodies not radiopaque on X-ray [9–11]. Compared to radiographs and ultrasound, non-contrast CT has improved sensitivity (~95%) [7].

There are several independent factors that play a role in the ability to identify a FB on ultrasound. Substances such as subcutaneous air or calcifications can obstruct the view or be mistaken for a FB. The detection sensitivity is also impacted by size, location, and scanning plane (parallel to the long axis) of the FB [9, 11]. Certain regions, especially in the hand and foot, can be more difficult to ultrasound considering their contour and close proximity to anatomical structures with varied sonographic appearances [11]. Technique modifications to improve acoustic windows, such as utilizing a water bath or standoff pad as the acoustic medium and scanning in multiple orientations, can improve visualization of both the foreign body and the artifacts they generate [11, 12]. The chemical composition of a foreign body induces different far field artifacts (i.e., metal and glass can induce reverberation artifacts) [13]. Organic matter can produce a circumferential hypoechoic halo secondary to inflammation/edema, abscess, or granulation tissue surrounding the foreign body and as a result helps in its localization [11, 13].

Lastly, a provider must consider the type of FB when choosing the modality to detect it. Ultrasound would be a better choice for compounds which are radiolucent and largely missed on radiography. In one study, radiography identified none of the foreign bodies which were radiolucent (wood, plastic, and organic material such as cactus spine) [9]. Wood, a common foreign body, has repeatedly been shown to be missed by plain radiography [11, 13, 14] with one study reporting a 7.4% sensitivity on plane radiograph [15]. A 2015 meta-analysis suggested that for ultrasound detection of a foreign body, the pooled sensitivity was 72% and specificity was 92% [12]. CT scans have a 5-15 times greater sensitivity than plain radiographs but are expensive, expose patients to high radiation burdens and may not be readily available in certain clinical environments [13]. These factors should lead providers to consider a multimodal approach when evaluating suspicious injuries for a retained foreign body. The order in which radiography and ultrasound are performed depends on the type of foreign body suspected, skill of the provider to perform the ultrasound, and availability of the imaging modalities. Those with easy access to point-of-care ultrasound should consider performing it as part of their initial evaluation but certainly if the radiography did not detect a foreign body when there is a high index of suspicion one may be present. Considering the overall success, plain radiograph and ultrasound should occur before performing a CT scan. CT can be considered the next imaging modality for foreign bodies that are not visualized on X-ray or ultrasound.

In our case report, ultrasound was able to identify the larger of the two foreign bodies noted on hand radiography and intraoperative exploration. This is likely secondary to the size and location of the foreign bodies. It is also necessary to consider operator skill, as this may have played a role in identifying only the larger FB. The findings, nonetheless, show the spine of the *Ariopsis felis* (hardhead catfish) can be visualized by bedside ultrasound and appear radiopaque on simple radiograph.

Surgery must be consulted to avoid causing further damage to the nearby structures and for potential surgical management under fluoroscopy. Intraoperative fluoroscopy is frequently helpful, as the objects can be difficult to locate in plain view (Figure 4). Once the foreign body is removed, the wound should be scrubbed and irrigated. Antibiotics and judicious pain management should be administered and the wound should be left open for prevention of secondary infections.

Appropriate antibiotic administration should cover gram negative rods with special consideration of Vibrio and Aeromonas species. Oral antibiotics such as ciprofloxacin or cephalosporins are preferred in the literature for both initial prophylactic treatment and treatment of active infection from catfish spine injuries. Tetanus prophylaxis should be given when indicated. Wound follow-up should be given within 1 week or sooner if systemic symptoms arise [2, 3].

### 3.1. Why Should an Emergency Physician Be Aware of This?

Penetrating injury from hardhead saltwater catfish dorsal spines can cause hidden foreign body, infection, and secondary damage to nearby structures. Imaging should be done for these patients to ensure they obtain timely and complete extraction of the venomous structures. Surgery must be consulted for operative management to avoid damage on removal of the catfish spine remnants.
